# Mercury Levels in Mothers

**DOI:** 10.1289/ehp.112-a978a

**Published:** 2004-12

**Authors:** Katherine M. Shea

**Affiliations:** Consultant, One Buttons Lane, Chapel Hill, North Carolina, E-mail: tkmjshea@mindspring.com

I read with great interest the excellent article by Mahaffey et al. (2004), which further describes the characteristics of the 1,709 women from the National Health and Nutrition Examination Survey (NHANES) 1999–2000 who were sampled for total and organic mercury levels in blood. It adds valuable detail to the initial report published last year (Schober et al. 2003). I would appreciate clarification on one important point: in the “Discussion,” the authors cited a new analysis which indicates that the cord blood:maternal blood ratio is not 1:1 as assumed by the National Research Council (NRC) in 2000 (Committee on the Toxicological Effects of Methylmercury 2000), but rather 1.7:1. Using the same benchmark dose lower limit and uncertainty factor used by the NRC, Mahaffey et al. (2004) calculated that blood total mercury levels > 3.5 μg/L in mothers could be associated with increased risk to the developing fetal nervous system. I am very interested in the details of this analysis and particularly in understanding why the uncertainty factor applied by the NRC to account in part for toxicokinetic variability does not compensate for uncertainty related to the cord blood:maternal blood mercury ratio. This is a critical concept because it has a dramatic impact on how many women may carry mercury levels in excess of what is believed to be safe for a fetus.

## References

[b1-ehp0112-a0978a] Committee on the Toxicological Effects of Methylmercury, Board on Environmental Studies and Toxicology, Commission on Life Sciences, National Research Council 2000. Toxicological Effects of Methylmercury. Washington, DC:National Academy Press. Available: http://www.nap.edu/books/0309071402/html/ [accessed 28 October 2004].

[b2-ehp0112-a0978a] Mahaffey KR, Clickner RP, Bodurow CC (2004). Blood organic mercury and dietary mercury intake: National Health and Nutrition Examination Survey, 1999 and 2000. Environ Health Perspect.

[b3-ehp0112-a0978a] Schober SE, Sinks TH, Jones RL, Bolger PM, McDowell M, Osterloh J (2003). Blood mercury levels in U.S. children and women of childbearing age, 1999–2000. JAMA.

